# {*N*,*N*′-Bis[1-(pyridin-2-yl)ethyl­idene]­propane-1,3-diamine}­(thio­cyanato-κ*N*)copper(II) tetra­fluoridoborate

**DOI:** 10.1107/S1600536811021040

**Published:** 2011-06-11

**Authors:** Li-Jun Liu

**Affiliations:** a Experimental Center, Linyi University, Linyi Shandong 276005, People’s Republic of China

## Abstract

In the title compound, [Cu(NCS)(C_17_H_20_N_4_)]BF_4_, the Cu^II^ ion is five-coordinated by the four N atoms of the tetra­dentate Schiff base ligand and one N atom of a thio­cyanate ligand, thereby forming a square-pyramidal CuN_5_ ccoordination geometry. The dihedral angle between the pyridine rings of the Schiff base is 55.58 (14)°. The F atoms of the tetra­fluoridoborate anion are disordered over two sets of sites with occupancies of 0.614 (3) and 0.386 (3). In the crystal, the components are linked by C—H⋯F inter­actions.

## Related literature

For background on the use of copper(II) complexes with Schiff bases in coordination chemistry and biological chemistry, see: Adhikary *et al.* (2009 ▶); Al-Karawi (2009 ▶); Xiao & Zhang (2009 ▶); Rajasekar *et al.* (2010 ▶); Sang & Lin (2010 ▶); Qin *et al.* (2010 ▶). For a related copper(II) complex that we reported recently, see: Liu (2010 ▶). For related copper complexes with square-pyramidal coordination, see: Liu *et al.* (1997 ▶); Chattopadhyay *et al.* (2006 ▶); Rahaman *et al.* (2005 ▶).
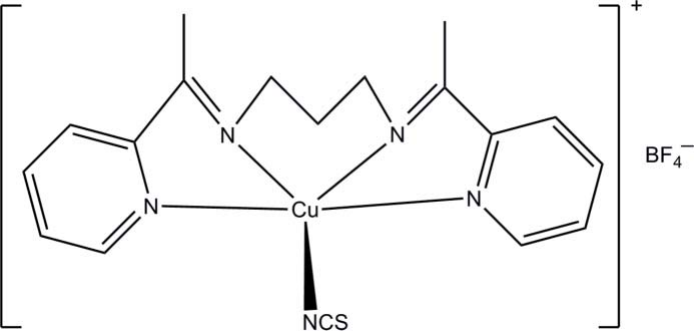


## Experimental

### 

#### Crystal data

[Cu(NCS)(C_17_H_20_N_4_)]BF_4_
*M* *_r_* = 488.80
Monoclinic, 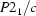

*a* = 9.5057 (7) Å
*b* = 13.7527 (11) Å
*c* = 16.1902 (13) Å
β = 101.200 (1)°
*V* = 2076.2 (3) Å^3^
*Z* = 4
Mo *K*α radiation
μ = 1.20 mm^−1^
*T* = 298 K
0.33 × 0.30 × 0.28 mm

#### Data collection

Bruker APEXII CCD diffractometer
Absorption correction: multi-scan (*SADABS*; Sheldrick, 2004 ▶) *T*_min_ = 0.692, *T*_max_ = 0.729
17908 measured reflections
5059 independent reflections
3368 reflections with *I* > 2σ(*I*)
*R*_int_ = 0.034

#### Refinement

*R*[*F* ^2^ > 2σ(*F* ^2^)] = 0.039
*wR*(*F* ^2^) = 0.107
*S* = 1.03
5059 reflections
310 parameters
56 restraints
H-atom parameters constrained
Δρ_max_ = 0.35 e Å^−3^
Δρ_min_ = −0.28 e Å^−3^

### 

Data collection: *APEX2* (Bruker, 2004 ▶); cell refinement: *SAINT* (Bruker, 2004 ▶); data reduction: *SAINT*; program(s) used to solve structure: *SHELXS97* (Sheldrick, 2008 ▶); program(s) used to refine structure: *SHELXL97* (Sheldrick, 2008 ▶); molecular graphics: *SHELXTL* (Sheldrick, 2008 ▶); software used to prepare material for publication: *SHELXTL*.

## Supplementary Material

Crystal structure: contains datablock(s) global, I. DOI: 10.1107/S1600536811021040/hb5899sup1.cif

Structure factors: contains datablock(s) I. DOI: 10.1107/S1600536811021040/hb5899Isup2.hkl

Additional supplementary materials:  crystallographic information; 3D view; checkCIF report

## Figures and Tables

**Table 1:** Selected bond lengths (Å)

Cu1—N2	1.986 (2)
Cu1—N3	2.003 (2)
Cu1—N4	2.021 (2)
Cu1—N1	2.063 (2)
Cu1—N5	2.091 (3)

**Table 2:** Hydrogen-bond geometry (Å, °)

*D*—H⋯*A*	*D*—H	H⋯*A*	*D*⋯*A*	*D*—H⋯*A*
C2—H2⋯F4^i^	0.93	2.49	3.416 (8)	176
C7—H7*B*⋯F4^i^	0.96	2.34	3.234 (6)	155

Symmetry code: (i) 
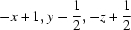
.

**Fig. 1.**
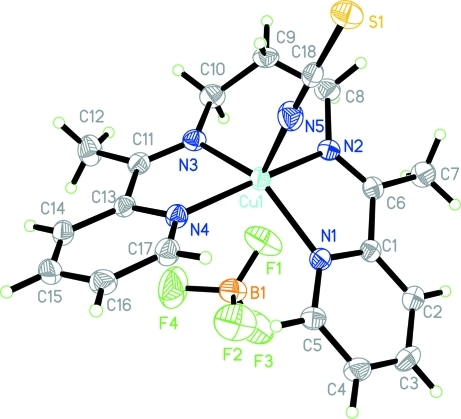
The molecular structure of the title complex with 30% probability displacement ellipsoids. Only the major component of the disordered fluoroborate group is shown.

## supplementary crystallographic information

### Comment

Copper(II) complexes with Schiff bases have been widely investigated in coordination chemistry and biological chemistry (Adhikary *et al.*, 2009; Al-Karawi, 2009; Xiao & Zhang, 2009; Rajasekar *et al.*, 2010; Sang & Lin, 2010; Qin *et al.*, 2010). As a continuation of our work on the Schiff base copper(II) complexes (Liu, 2010), in the present paper, the title new copper complex is reported.

The title compound contains a mononuclear copper(II) complex cation and a disordered fluoroborate anion, Fig. 1. The Cu^II^ atom in the complex is five-coordinated by the four N atoms of the Schiff base ligand, and by one N atom of a thiocyanate ligand, forming a square-pyramidal geometry. The bond lengths (Table 1) related to the Cu atom are comparable with those observed in similar copper complexes with square-pyramidal geometry (Liu *et al.*, 1997; Chattopadhyay *et al.*, 2006; Rahaman *et al.*, 2005).

### Experimental

2-Acetylpyridine (0.2 mmol, 24.2 mg), propane-1,3-diamine (0.1 mmol, 7.4 mg), ammonium thiocyanate (0.1 mmol, 7.6 mg), copper acetate (0.1 mmol, 19.9 mg), and ammonium fluoroborate (0.1 mmol, 10.5 mg) were mixed and stirred in methanol (20 ml) at reflux for 2 h, to give a blue solution. The solution was cooled to room temperature, and blue block-shaped single crystals were formed by slow evaporation of the solution in air.

### Refinement

H atoms were positioned geometrically (C–H = 0.93–0.97 Å) and refined using a riding model, with *U*_iso_(H) = 1.2*U*_eq_(C) and 1.5*U*_eq_(C_methyl_). The fluoroborate anion is disordered over two sites, with occupancies of 0.614 (3) and 0.386 (3).

### Crystal data

**Table:**

[Cu(NCS)(C_17_H_20_N_4_)]BF_4_	*F*(000) = 996
*M**_r_* = 488.80	*D*_x_ = 1.564 Mg m^−^^3^
Monoclinic, *P*2_1_/*c*	Mo *K*α radiation, λ = 0.71073 Å
*a* = 9.5057 (7) Å	Cell parameters from 4043 reflections
*b* = 13.7527 (11) Å	θ = 2.5–25.1°
*c* = 16.1902 (13) Å	µ = 1.20 mm^−^^1^
β = 101.200 (1)°	*T* = 298 K
*V* = 2076.2 (3) Å^3^	Block, blue
*Z* = 4	0.33 × 0.30 × 0.28 mm

### Data collection

**Table:**

Bruker APEXII CCD diffractometer	5059 independent reflections
Radiation source: fine-focus sealed tube	3368 reflections with *I* > 2σ(*I*)
graphite	*R*_int_ = 0.034
ω scans	θ_max_ = 28.3°, θ_min_ = 2.6°
Absorption correction: multi-scan (*SADABS*; Sheldrick, 2004)	*h* = −10→12
*T*_min_ = 0.692, *T*_max_ = 0.729	*k* = −18→18
17908 measured reflections	*l* = −21→21

### Refinement

**Table:**

Refinement on *F*^2^	Primary atom site location: structure-invariant direct methods
Least-squares matrix: full	Secondary atom site location: difference Fourier map
*R*[*F*^2^ > 2σ(*F*^2^)] = 0.039	Hydrogen site location: inferred from neighbouring sites
*wR*(*F*^2^) = 0.107	H-atom parameters constrained
*S* = 1.03	*w* = 1/[σ^2^(*F*_o_^2^) + (0.0515*P*)^2^ + 0.3601*P*] where *P* = (*F*_o_^2^ + 2*F*_c_^2^)/3
5059 reflections	(Δ/σ)_max_ = 0.001
310 parameters	Δρ_max_ = 0.35 e Å^−^^3^
56 restraints	Δρ_min_ = −0.28 e Å^−^^3^

### Special details

**Table:**

Geometry. All e.s.d.'s (except the e.s.d. in the dihedral angle between two l.s. planes) are estimated using the full covariance matrix. The cell e.s.d.'s are taken into account individually in the estimation of e.s.d.'s in distances, angles and torsion angles; correlations between e.s.d.'s in cell parameters are only used when they are defined by crystal symmetry. An approximate (isotropic) treatment of cell e.s.d.'s is used for estimating e.s.d.'s involving l.s. planes.
Refinement. Refinement of *F*^2^ against ALL reflections. The weighted *R*-factor *wR* and goodness of fit *S* are based on *F*^2^, conventional *R*-factors *R* are based on *F*, with *F* set to zero for negative *F*^2^. The threshold expression of *F*^2^ > σ(*F*^2^) is used only for calculating *R*-factors(gt) *etc*. and is not relevant to the choice of reflections for refinement. *R*-factors based on *F*^2^ are statistically about twice as large as those based on *F*, and *R*- factors based on ALL data will be even larger.

### Fractional atomic coordinates and isotropic or equivalent isotropic displacement parameters (Å^2^)

**Table:**

	*x*	*y*	*z*	*U*_iso_*/*U*_eq_	Occ. (<1)
Cu1	0.86559 (4)	0.35520 (2)	0.20844 (2)	0.04286 (12)	
S1	1.20466 (9)	0.30423 (6)	0.02388 (5)	0.0610 (2)	
N1	0.8455 (2)	0.22227 (15)	0.26403 (13)	0.0417 (5)	
N2	0.7105 (2)	0.29716 (16)	0.12272 (13)	0.0443 (5)	
N3	0.7727 (2)	0.48651 (16)	0.19522 (13)	0.0445 (5)	
N4	0.9890 (2)	0.42656 (15)	0.30527 (13)	0.0431 (5)	
N5	1.0234 (3)	0.33564 (18)	0.13614 (17)	0.0594 (7)	
C1	0.7394 (3)	0.16805 (18)	0.21868 (16)	0.0409 (6)	
C2	0.7017 (3)	0.0789 (2)	0.24686 (18)	0.0506 (7)	
H2	0.6281	0.0427	0.2149	0.061*	
C3	0.7750 (4)	0.0439 (2)	0.3233 (2)	0.0594 (8)	
H3	0.7513	−0.0160	0.3435	0.071*	
C4	0.8819 (4)	0.0983 (2)	0.36833 (19)	0.0593 (8)	
H4	0.9333	0.0757	0.4195	0.071*	
C5	0.9139 (3)	0.1878 (2)	0.33747 (18)	0.0523 (7)	
H5	0.9861	0.2253	0.3694	0.063*	
C6	0.6669 (3)	0.21236 (19)	0.13808 (16)	0.0424 (6)	
C7	0.5498 (3)	0.1593 (2)	0.0812 (2)	0.0595 (8)	
H7A	0.4592	0.1888	0.0838	0.089*	
H7B	0.5491	0.0926	0.0986	0.089*	
H7C	0.5656	0.1623	0.0244	0.089*	
C8	0.6416 (4)	0.3483 (2)	0.04519 (18)	0.0578 (8)	
H8A	0.5383	0.3446	0.0393	0.069*	
H8B	0.6676	0.3166	−0.0032	0.069*	
C9	0.6869 (3)	0.4539 (2)	0.04699 (17)	0.0540 (7)	
H9A	0.7885	0.4569	0.0459	0.065*	
H9B	0.6358	0.4856	−0.0035	0.065*	
C10	0.6597 (3)	0.5092 (2)	0.12256 (17)	0.0572 (8)	
H10A	0.6588	0.5785	0.1112	0.069*	
H10B	0.5670	0.4912	0.1345	0.069*	
C11	0.8076 (3)	0.54422 (19)	0.25782 (17)	0.0455 (6)	
C12	0.7350 (4)	0.6387 (2)	0.2680 (2)	0.0727 (10)	
H12A	0.7947	0.6915	0.2568	0.109*	
H12B	0.7187	0.6439	0.3245	0.109*	
H12C	0.6448	0.6414	0.2291	0.109*	
C13	0.9330 (3)	0.51342 (18)	0.32143 (16)	0.0421 (6)	
C14	0.9964 (3)	0.5691 (2)	0.38931 (17)	0.0520 (7)	
H14	0.9556	0.6280	0.4002	0.062*	
C15	1.1200 (3)	0.5373 (2)	0.44084 (18)	0.0569 (8)	
H15	1.1627	0.5738	0.4874	0.068*	
C16	1.1795 (3)	0.4511 (2)	0.42266 (18)	0.0564 (8)	
H16	1.2640	0.4286	0.4560	0.068*	
C17	1.1114 (3)	0.3979 (2)	0.35371 (19)	0.0526 (7)	
H17	1.1529	0.3401	0.3408	0.063*	
C18	1.0978 (3)	0.32353 (18)	0.08884 (17)	0.0418 (6)	
B1	0.5510 (4)	0.3543 (3)	0.3428 (2)	0.0678 (11)	0.614 (8)
F1	0.5253 (15)	0.3475 (8)	0.2600 (4)	0.138 (4)	0.614 (8)
F2	0.6776 (6)	0.3143 (6)	0.3830 (3)	0.130 (3)	0.614 (8)
F3	0.4436 (7)	0.3062 (7)	0.3717 (3)	0.140 (3)	0.614 (8)
F4	0.5594 (10)	0.4452 (3)	0.3778 (4)	0.153 (3)	0.614 (8)
B1'	0.5510 (4)	0.3543 (3)	0.3428 (2)	0.0678 (11)	0.386 (8)
F1'	0.527 (2)	0.3819 (11)	0.2591 (5)	0.116 (4)	0.386 (8)
F2'	0.5547 (12)	0.2555 (4)	0.3521 (5)	0.110 (3)	0.386 (8)
F3'	0.4465 (10)	0.3895 (9)	0.3752 (5)	0.112 (4)	0.386 (8)
F4'	0.6822 (10)	0.3896 (10)	0.3720 (6)	0.140 (5)	0.386 (8)

### Atomic displacement parameters (Å^2^)

**Table:**

	*U*^11^	*U*^22^	*U*^33^	*U*^12^	*U*^13^	*U*^23^
Cu1	0.0456 (2)	0.03860 (18)	0.0420 (2)	0.00179 (14)	0.00258 (14)	−0.00137 (13)
S1	0.0590 (5)	0.0726 (5)	0.0545 (5)	0.0038 (4)	0.0188 (4)	0.0015 (4)
N1	0.0429 (13)	0.0401 (11)	0.0416 (12)	0.0034 (10)	0.0073 (10)	0.0000 (9)
N2	0.0466 (14)	0.0464 (13)	0.0386 (12)	0.0007 (10)	0.0055 (10)	0.0018 (10)
N3	0.0445 (13)	0.0446 (12)	0.0440 (12)	0.0077 (10)	0.0078 (10)	0.0055 (10)
N4	0.0427 (13)	0.0398 (11)	0.0458 (12)	0.0009 (10)	0.0059 (10)	−0.0008 (9)
N5	0.0611 (17)	0.0551 (15)	0.0660 (17)	−0.0031 (13)	0.0226 (15)	−0.0104 (13)
C1	0.0391 (15)	0.0404 (14)	0.0455 (15)	0.0049 (11)	0.0141 (12)	−0.0029 (11)
C2	0.0499 (17)	0.0439 (15)	0.0594 (18)	−0.0006 (13)	0.0140 (14)	−0.0017 (13)
C3	0.066 (2)	0.0422 (16)	0.072 (2)	0.0067 (15)	0.0195 (17)	0.0127 (15)
C4	0.066 (2)	0.0563 (18)	0.0538 (18)	0.0100 (16)	0.0063 (15)	0.0111 (14)
C5	0.0571 (19)	0.0519 (16)	0.0443 (15)	0.0035 (14)	0.0012 (13)	0.0039 (13)
C6	0.0376 (15)	0.0458 (15)	0.0449 (15)	0.0022 (12)	0.0109 (12)	−0.0044 (12)
C7	0.0503 (19)	0.0624 (19)	0.0613 (19)	−0.0083 (15)	−0.0005 (15)	0.0012 (15)
C8	0.059 (2)	0.0634 (19)	0.0449 (16)	−0.0037 (15)	−0.0039 (14)	0.0073 (14)
C9	0.0571 (19)	0.0591 (18)	0.0427 (15)	0.0018 (14)	0.0020 (13)	0.0125 (13)
C10	0.0585 (19)	0.0557 (17)	0.0535 (17)	0.0154 (15)	0.0006 (15)	0.0094 (14)
C11	0.0482 (16)	0.0412 (14)	0.0502 (16)	0.0020 (12)	0.0171 (13)	0.0019 (12)
C12	0.077 (2)	0.0548 (19)	0.084 (2)	0.0179 (17)	0.009 (2)	−0.0135 (17)
C13	0.0466 (16)	0.0378 (13)	0.0450 (14)	−0.0036 (11)	0.0163 (12)	−0.0003 (11)
C14	0.065 (2)	0.0424 (14)	0.0514 (17)	−0.0083 (14)	0.0187 (15)	−0.0076 (13)
C15	0.066 (2)	0.0581 (18)	0.0460 (16)	−0.0184 (16)	0.0084 (15)	−0.0048 (14)
C16	0.0533 (18)	0.0627 (19)	0.0493 (16)	−0.0093 (15)	0.0002 (14)	0.0076 (14)
C17	0.0503 (18)	0.0466 (15)	0.0583 (18)	0.0014 (13)	0.0039 (14)	−0.0017 (13)
C18	0.0446 (16)	0.0311 (12)	0.0472 (15)	−0.0029 (11)	0.0025 (13)	0.0000 (11)
B1	0.072 (3)	0.071 (3)	0.064 (3)	0.023 (2)	0.021 (2)	0.008 (2)
F1	0.180 (6)	0.172 (8)	0.065 (4)	−0.003 (6)	0.029 (4)	−0.006 (3)
F2	0.109 (5)	0.147 (6)	0.138 (4)	0.076 (5)	0.034 (4)	0.016 (4)
F3	0.116 (5)	0.208 (8)	0.098 (3)	−0.066 (6)	0.027 (3)	−0.015 (4)
F4	0.190 (7)	0.075 (3)	0.175 (5)	0.035 (4)	−0.011 (4)	−0.017 (3)
B1'	0.072 (3)	0.071 (3)	0.064 (3)	0.023 (2)	0.021 (2)	0.008 (2)
F1'	0.142 (7)	0.129 (8)	0.084 (7)	0.010 (6)	0.038 (5)	0.041 (5)
F2'	0.128 (7)	0.077 (4)	0.122 (5)	0.005 (4)	0.013 (5)	0.011 (4)
F3'	0.111 (7)	0.145 (7)	0.094 (5)	0.073 (6)	0.059 (4)	0.020 (5)
F4'	0.114 (7)	0.169 (8)	0.127 (6)	−0.055 (7)	0.001 (5)	0.000 (6)

### Geometric parameters (Å, °)

**Table:**

Cu1—N2	1.986 (2)	C7—H7C	0.9600
Cu1—N3	2.003 (2)	C8—C9	1.513 (4)
Cu1—N4	2.021 (2)	C8—H8A	0.9700
Cu1—N1	2.063 (2)	C8—H8B	0.9700
Cu1—N5	2.091 (3)	C9—C10	1.505 (4)
S1—C18	1.620 (3)	C9—H9A	0.9700
N1—C5	1.327 (3)	C9—H9B	0.9700
N1—C1	1.351 (3)	C10—H10A	0.9700
N2—C6	1.278 (3)	C10—H10B	0.9700
N2—C8	1.477 (3)	C11—C13	1.478 (4)
N3—C11	1.279 (3)	C11—C12	1.496 (4)
N3—C10	1.464 (3)	C12—H12A	0.9600
N4—C17	1.330 (3)	C12—H12B	0.9600
N4—C13	1.354 (3)	C12—H12C	0.9600
N5—C18	1.151 (4)	C13—C14	1.378 (4)
C1—C2	1.380 (4)	C14—C15	1.373 (4)
C1—C6	1.483 (4)	C14—H14	0.9300
C2—C3	1.383 (4)	C15—C16	1.371 (4)
C2—H2	0.9300	C15—H15	0.9300
C3—C4	1.355 (4)	C16—C17	1.385 (4)
C3—H3	0.9300	C16—H16	0.9300
C4—C5	1.385 (4)	C17—H17	0.9300
C4—H4	0.9300	B1—F1	1.320 (7)
C5—H5	0.9300	B1—F2	1.367 (5)
C6—C7	1.491 (4)	B1—F4	1.368 (5)
C7—H7A	0.9600	B1—F3	1.372 (5)
C7—H7B	0.9600		
			
N2—Cu1—N3	92.05 (9)	N2—C8—H8A	109.4
N2—Cu1—N4	167.96 (9)	C9—C8—H8A	109.4
N3—Cu1—N4	79.77 (9)	N2—C8—H8B	109.4
N2—Cu1—N1	80.19 (9)	C9—C8—H8B	109.4
N3—Cu1—N1	140.31 (9)	H8A—C8—H8B	108.0
N4—Cu1—N1	100.42 (8)	C10—C9—C8	114.0 (3)
N2—Cu1—N5	94.19 (10)	C10—C9—H9A	108.7
N3—Cu1—N5	113.90 (9)	C8—C9—H9A	108.7
N4—Cu1—N5	97.20 (9)	C10—C9—H9B	108.7
N1—Cu1—N5	105.51 (9)	C8—C9—H9B	108.7
C5—N1—C1	118.3 (2)	H9A—C9—H9B	107.6
C5—N1—Cu1	129.53 (19)	N3—C10—C9	109.3 (2)
C1—N1—Cu1	112.00 (17)	N3—C10—H10A	109.8
C6—N2—C8	119.6 (2)	C9—C10—H10A	109.8
C6—N2—Cu1	117.11 (18)	N3—C10—H10B	109.8
C8—N2—Cu1	123.28 (18)	C9—C10—H10B	109.8
C11—N3—C10	122.8 (2)	H10A—C10—H10B	108.3
C11—N3—Cu1	115.70 (18)	N3—C11—C13	115.1 (2)
C10—N3—Cu1	121.13 (18)	N3—C11—C12	124.9 (3)
C17—N4—C13	118.6 (2)	C13—C11—C12	120.0 (3)
C17—N4—Cu1	128.55 (19)	C11—C12—H12A	109.5
C13—N4—Cu1	112.82 (17)	C11—C12—H12B	109.5
C18—N5—Cu1	172.4 (3)	H12A—C12—H12B	109.5
N1—C1—C2	121.7 (2)	C11—C12—H12C	109.5
N1—C1—C6	115.0 (2)	H12A—C12—H12C	109.5
C2—C1—C6	123.3 (3)	H12B—C12—H12C	109.5
C1—C2—C3	119.1 (3)	N4—C13—C14	121.1 (3)
C1—C2—H2	120.4	N4—C13—C11	114.3 (2)
C3—C2—H2	120.4	C14—C13—C11	124.4 (2)
C4—C3—C2	119.0 (3)	C15—C14—C13	119.8 (3)
C4—C3—H3	120.5	C15—C14—H14	120.1
C2—C3—H3	120.5	C13—C14—H14	120.1
C3—C4—C5	119.4 (3)	C16—C15—C14	119.0 (3)
C3—C4—H4	120.3	C16—C15—H15	120.5
C5—C4—H4	120.3	C14—C15—H15	120.5
N1—C5—C4	122.5 (3)	C15—C16—C17	118.8 (3)
N1—C5—H5	118.8	C15—C16—H16	120.6
C4—C5—H5	118.8	C17—C16—H16	120.6
N2—C6—C1	115.7 (2)	N4—C17—C16	122.5 (3)
N2—C6—C7	123.9 (2)	N4—C17—H17	118.8
C1—C6—C7	120.4 (2)	C16—C17—H17	118.8
C6—C7—H7A	109.5	N5—C18—S1	178.5 (3)
C6—C7—H7B	109.5	F1—B1—F2	115.3 (7)
H7A—C7—H7B	109.5	F1—B1—F4	118.0 (6)
C6—C7—H7C	109.5	F2—B1—F4	101.3 (5)
H7A—C7—H7C	109.5	F1—B1—F3	108.0 (7)
H7B—C7—H7C	109.5	F2—B1—F3	106.8 (5)
N2—C8—C9	111.3 (2)	F4—B1—F3	106.7 (5)

### Hydrogen-bond geometry (Å, °)

**Table:**

*D*—H···*A*	*D*—H	H···*A*	*D*···*A*	*D*—H···*A*
C2—H2···F4^i^	0.93	2.49	3.416 (8)	176
C7—H7B···F4^i^	0.96	2.34	3.234 (6)	155

Symmetry codes: (i) −*x*+1, *y*−1/2, −*z*+1/2.
